# Upregulation of miR-24 is associated with a decreased DNA damage response upon etoposide treatment in highly differentiated CD8^+^ T cells sensitizing them to apoptotic cell death

**DOI:** 10.1111/j.1474-9726.2012.00819.x

**Published:** 2012-08

**Authors:** Stefan Brunner, Dietmar Herndler-Brandstetter, Christoph R Arnold, Gerrit Jan Wiegers, Andreas Villunger, Matthias Hackl, Johannes Grillari, María Moreno-Villanueva, Alexander Bürkle, Beatrix Grubeck-Loebenstein

**Affiliations:** 1 Immunology Division, Institute for Biomedical Aging Research, Austrian Academy of SciencesInnsbruck, Austria; 2 Division of Developmental Immunology, Biocenter, University of InnsbruckInnsbruck, Austria; 3 Department of Biotechnology, Aging and Immortalization Research, University of Natural Resources and Applied Life SciencesVienna, Austria; 4 Molecular Toxicology Group, University of KonstanzKonstanz, Germany

**Keywords:** aging, DNA damage, DNA repair, senescence, CD8^+^ T cells, apoptosis, etoposide

## Abstract

The life-long homeostasis of memory CD8^+^ T cells as well as persistent viral infections have been shown to facilitate the accumulation of highly differentiated CD8^+^CD28^−^ T cells, a phenomenon that has been associated with an impaired immune function in humans. However, the molecular mechanisms regulating homeostasis of CD8^+^CD28^−^ T cells have not yet been elucidated. In this study, we demonstrate that the miR-23∼24∼27 cluster is up-regulated during post-thymic CD8^+^ T-cell differentiation in humans. The increased expression of miR-24 in CD8^+^CD28^−^ T cells is associated with decreased expression of the histone variant H2AX, a protein that plays a key role in the DNA damage response (DDR). Following treatment with the classic chemotherapeutic agent etoposide, a topoisomerase II inhibitor, apoptosis was increased in CD8^+^CD28^−^ when compared to CD8^+^CD28^+^ T cells and correlated with an impaired DDR in this cell type. The reduced capacity of CD8^+^CD28^−^ T cell to repair DNA was characterized by the automated fluorimetric analysis of DNA unwinding (FADU) assay as well as by decreased phosphorylation of H2AX at Ser139, of ATM at Ser1981, and of p53 at Ser15. Interleukin (IL)-15 could prevent etoposide-mediated apoptosis of CD8^+^CD28^−^ T cells, suggesting a role for IL-15 in the survival and the age-dependent accumulation of CD8^+^CD28^−^ T cells in humans.

## Introduction

Human aging is associated with the involution of the thymus (Steinmann *et al.*, 1985), thereby narrowing the T-cell repertoire in old age. While the number of naive T cells decreases, memory and effector T cells accumulate (Arnold *et al.*, 2011). These changes are particularly pronounced in the CD8^+^ T-cell pool (Saule *et al.*, 2006). The increase in the number of highly differentiated CD8^+^CD28^−^ effector T cells has attracted more attention over the last decade. This specific cell type dominates the repertoire in elderly persons and is especially high in elderly persons who also have persistent infection with cytomegalovirus (CMV) (Brunner *et al.*, 2011). High numbers of CD8^+^CD28^−^ T cells are believed to be deleterious for elderly persons, as their accumulation has been linked to a shortened live expectancy, enhanced progression of age-related diseases such as Alzheimer’s disease and atherosclerosis as well as with a low efficacy of vaccination (Goronzy *et al.*, 2001; Saurwein-Teissl *et al.*, 2002; Wikby *et al.*, 2005). For these reasons, therapeutic elimination of this particular cell population has been considered (Pawelec *et al.*, 2006). However, potential treatment regimes to reach this goal depend on a clear understanding of the way how CD8^+^CD28^−^ T cells are generated, survive, and are eliminated *in vivo*. Presently, there is more controversy on these issues. While some claim that terminally differentiated T cells are particularly prone to undergo apoptosis and are therefore short-lived, others suggest that they are long-lived, probably due to an intrinsic resistance to apoptosis-inducing stimuli (Spaulding *et al.*, 1999; Effros *et al.*, 2005; Wallace *et al.*, 2011).

Recent results from our laboratory demonstrate that the microRNA (miRNA) expression pattern differs between CD8^+^CD28^+^ and CD8^+^CD28^−^ T cells (Hackl *et al.*, 2010). Of particular interest in this context is a decreased expression of the polycistronic miRNA cluster miR-17∼92 in CD8^+^CD28^−^ T cells, which was also found to be down-regulated in other human cell types following multiple rounds of division. Of note, this miRNA cluster is known to target the cyclin-dependent kinase inhibitor p21 as well as the pro-apoptotic B-cell lymphoma 2 (BCL_2_) family protein Bim, critical for lymphocyte homeostasis (van Haaften & Agami, 2010). miRNAs might therefore be of importance for replicative exhaustion by interfering with important mediators of the cell cycle and apoptosis (Grillari *et al.*, 2010). In the course of our previous experiments, another miRNA cluster was noticed to be overexpressed in CD8^+^CD28^−^ T cells, but not in the other cell types studied.

This miRNA cluster, miR-23∼24∼27, has been shown to target molecules important for DNA repair (Lal *et al.*, 2009; Srivastava *et al.*, 2011). Decreased DNA repair during cellular senescence as well as in parallel to T-cell differentiation has previously been reported (Scarpaci *et al.*, 2003; Seluanov *et al.*, 2004).

We therefore wondered whether the DNA damage response (DDR) might be impaired in CD8^+^CD28^−^ T cells, leading to an increased susceptibility to apoptosis in this cell type. To investigate this possibility, we further analyzed miRNA expression profiles, DNA repair, and apoptosis in CD8^+^CD28^+^ and CD8^+^CD28^−^ T cells. We noted decreased expression of the histone H2A family member X (H2AX), a validated target of miR-24 (Lal *et al.*, 2009; Srivastava *et al.*, 2011), in CD8^+^CD28^−^ T cells. This impairment was associated with changes in DDR signaling events, with persisting DNA strand breaks (DSBs), as well as with an increased occurrence of apoptosis in CD8^+^CD28^−^ T cells following induced DNA damage that could be prevented by interleukin (IL)-15. We therefore propose that the increased vulnerability of CD8^+^CD28^−^ T cells to apoptotic cell death is balanced by IL-15, supporting their survival in IL-15-rich niches such as the bone marrow (BM) (Herndler-Brandstetter *et al.*, 2011a, b).

## Results

### miRNA expression profiling of CD8^+^ T-cell subsets

**Fig. 1:**
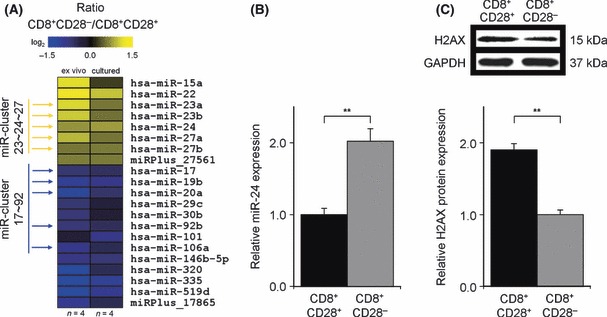
(A) Global miRNA expression profile of human CD8^+^CD28^+^ and CD8^+^CD28^−^ T cells. CD8^+^ T-cell subsets were analyzed directly *ex vivo* or cultured for 16 h: consistently regulated miRNAs under each experimental condition are shown on a log_2_ scale (yellow = up-regulated, blue = down-regulated in CD8^+^CD28^−^ compared to CD8^+^CD28^+^ T cells). The heatmap represents mean ratios of four experiments with cells from individual donors; mean log_2_ regulation; FDR-adjusted *P* ≤ 0.05; CD8^+^CD28^−^ versus CD8^+^CD28^+^. (B) Quantification of miR-24 levels in CD8^+^CD28^+^ and CD8^+^CD28^−^ T cells by RT–PCR. The bar graph shows the relative miR-24 expression normalized to GAPDH; mean fold-ratios ± SEM; *n* = 5; **P ≤ 0.01; CD8^+^CD28^−^ versus CD8^+^CD28^+^. (C) H2AX expression in CD8^+^CD28^+^ and CD8^+^CD28^−^ T cells. The histone H2A variant H2AX was assessed in freshly isolated CD8^+^ T-cell subsets by Western Blot analysis of which one representative example of five is shown. The bar graph shows the densitometrical evaluation of grayscale values normalized to GAPDH; mean fold-ratios ± SEM; *n* = 5; **P ≤ 0.01; CD8^+^CD28^+^ versus CD8^+^CD28^−^.

We first compared the global miRNA expression profiles of CD8^+^CD28^+^ and CD8^+^CD28^−^ T cells. A log_2_-corrected heatmap of all 21 regulated miRNAs is depicted in Fig. 1A. This screening approach revealed a pronounced dysregulation of two miRNA clusters in CD8^+^CD28^−^ T cells that is consistent over a 16-h period of cell culture (second column in the heatmap), a control used to test the effect of cell culture conditions on miRNA expression. These miRNA clusters are miR-17∼92 and miR-23∼24∼27, the members of which are cleaved out of a polycistronic pri-miRNA-variant. Compared to CD8^+^CD28^+^ T cells, CD8^+^CD28^−^ T cells express less miR-17, miR-19b, miR-20a, miR-92b, and miR-106a, all of which are members of the paralogous miRNA clusters miR-17∼92, miR-106a∼363, and miR-106b∼25 with extensive sequence homologies. This miRNA cluster is not only down-regulated in highly differentiated CD8^+^CD28^−^ compared to CD8^+^CD28^+^ T cells, but also in other models of replicative or chronological aging (Hackl *et al.*, 2010).

**Fig. 2:**
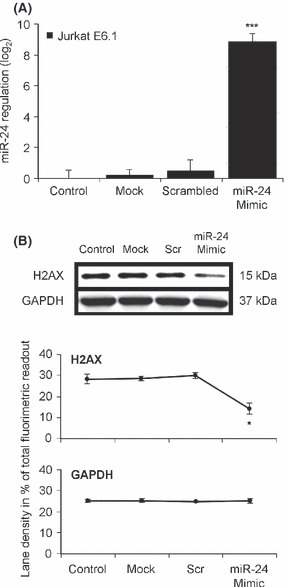
(A) miR-24 overexpression in the human leukemic T-cell lymphoblast line Jurkat E6.1. Cells were either left untreated (Control) or transfected with empty (Mock), nonsense miRNA (Scrambled), or miR-24 Mimic-filled transfection complexes. The bar graph shows the relative miR-24 regulation normalized to GAPDH 48-h post-transfection; mean log_2_-ratios ± SEM; *n* = 4; ***P ≤ 0.001; miR-24 Mimic versus controls. (B) H2AX protein expression in miR-24 transfected Jurkat cells 96-h post-transfection. The H2AX content was assessed by Western Blot analysis of which one of four representative images is shown. The graph shows the densitometrical evaluation of grayscale values. Data represent fluorescence in individual lanes as percentages of total fluorescence in the whole blot; means ± SEM; *n* = 4; *P ≤ 0.05; miR-24 Mimic versus controls.

The second miRNA cluster, which is differentially expressed in CD8^+^CD28^−^ T cells, is the miR-23∼24∼27 cluster. Its members were up-regulated in CD8^+^CD28^−^ in comparison with CD8^+^CD28^+^ T cells by factors between 1.5 and 2.9. The regulation of each miRNA variant in this cluster was statistically significant (*P* adj. ≤ 0.001). We were particularly interested in the regulation of miR-24, as it targets the H2AX, an important mediator of DSB repair (Lal *et al.*, 2009; Srivastava *et al.*, 2011). We therefore validated our array data on miR-24 by quantitative RT–PCR (Fig. 1B). We also analyzed H2AX protein expression in the two CD8^+^ T-cell subsets. Densitometric evaluation of Western Blot data, as shown in Fig. 1C, demonstrates a decreased H2AX protein expression in CD8^+^CD28^−^ T cells in comparison with their CD8^+^CD28^+^ T-cell counterparts. To confirm H2AX as a cellular target of miR-24, we overexpressed miR-24 in the lymphoblast T-cell line Jurkat E6.1. Forty-eight-hour post-transfection miR-24 levels were strongly up-regulated (Fig. 2). This led to a significant down-regulation of H2AX protein compared to controls 96-h post-transfection (Fig. 2).

### DDR signaling in CD8^+^ T-cell subsets

**Fig. 3:**
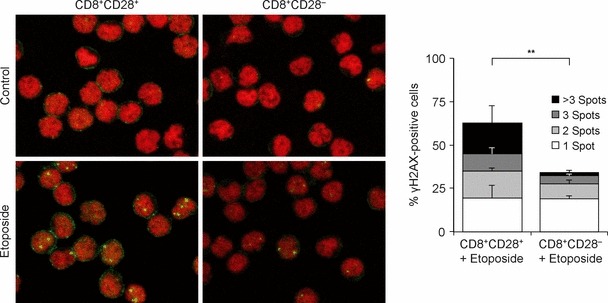
Initiation of DNA damage response in human CD8^+^CD28^+^ and CD8^+^CD28^−^ T cells following etoposide treatment. Immunofluorescence stainings of DNA (TO-PRO, red) and phosphorylated γH2AX at Ser139 (green) in CD8^+^CD28^+^ and CD8^+^CD28^−^ T cells after 30-minute exposure to etoposide (10 μg mL^−1^) were analyzed by confocal microscopy. One representative set of images from one donor is shown per CD8^+^ T-cell subset and treatment. The bar graph indicates γH2AX spot-positive cells with the absolute number of spots in the nuclei of the cells indicated; means in % of total cells ± SEM; *n* = 4; **P ≤ 0.01.

**Fig. 4:**
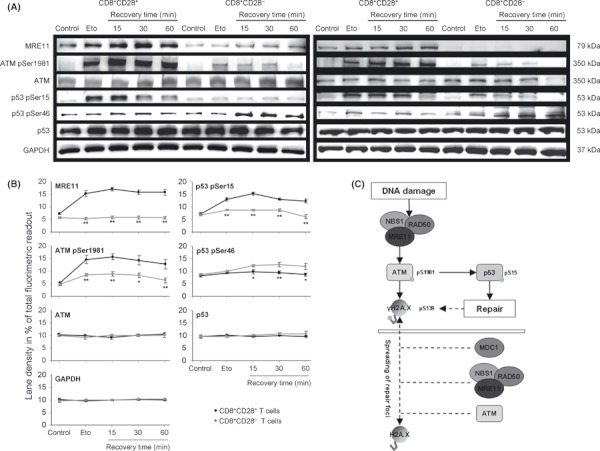
(A) DNA damage response (DDR) signaling in human CD8^+^CD28^+^ and CD8^+^CD28^−^ T cells after etoposide exposure. Isolated CD8^+^CD28^+^ and CD8^+^CD28^−^ T cells of elderly (≥ 65 years, left panel) and young (≤ 30 years, right panel) donors were left as untreated control or treated with 10 μg mL^−1^ etoposide for 60 min. After etoposide removal, the T-cell subsets were given time to repair the induced DNA damage for 15, 30, and 60 min at 37 °C and 5% CO_2_. Regulation of essential components of the DDR machinery and apoptosis induction were analyzed by Western Blot. Data are representative of experiments with cells from elderly (*n* = 5) and young (*n* = 3) donors. (B) The graph shows the densitometrical evaluation of grayscale values. Data represent fluorescence in individual lanes as percentages of total fluorescence in the whole blot; means ± SEM; *n* = 8, as there was no difference between cells from young and old donors, results from both age groups were included in the calculation; *P ≤ 0.05; **P ≤ 0.01; CD8^+^CD28^+^ (black) versus CD8^+^CD28^−^ (gray). (C) Schematic representation of early DDR signaling events. The MRN nuclease/helicase complex, consisting of MRE11, RAD50, and NBS1, initially senses DNA strand breaks and recruits and activates monomeric ataxia telangiectasia mutated (ATM) by phosphorylation (Ser1981). At the site of DNA damage, ATM initiates the phosphorylation of H2AX (Ser139) to generate γH2AX, which elicits a sequence of signaling events that feed into a positive feedback loop to recruit more enzymes (Sengupta & Harris, 2005). Hereby, γH2AX serves as a landing pad for the retention of further essential components of the DNA repair machinery, especially MDC1 (mediator of DNA damage checkpoint 1) as well as further MRN complexes and phospho-ATM molecules, to spread and reach more distal chromatin regions (West & van Attikum, 2006).

In a next step, we analyzed whether the reduced expression of H2AX in CD8^+^CD28^−^ T cells is associated with changes in the DDR in highly differentiated CD8^+^ T cells. As a model to induce DNA damage, isolated CD8^+^ T-cell subsets were exposed to etoposide, a potent topoisomerase II inhibitor that interferes in the biphasic reaction of this ubiquitous enzyme and prohibits the re-ligation of previously introduced DSBs (Baldwin & Osheroff, 2005). As one of the earliest events in the DDR pathway is the phosphorylation of H2AX at Ser139 (γH2AX), we performed immunofluorescence staining of γH2AX on etoposide-treated CD8^+^CD28^+^ and CD8^+^CD28^−^ T cells. Our results demonstrate intact H2AX phosphorylation and γH2AX cluster formation in nuclei of CD8^+^CD28^+^ T cells, while CD8^+^CD28^−^ T cells failed to mount such a response properly, both in terms of the percentage of cells with γH2AX clusters in their nuclei as well as the numbers of clusters in the nuclei of individual cells (Fig. 3). These results indicate an impaired DDR in highly differentiated CD8^+^ T cells. We next elucidated the regulation of important signaling events in the cascade of the DDR. Therefore, phosphorylated (Ser1981) ataxia telangiectasia mutated (ATM) protein, one of the first sensors of DNA damage, phosphorylated (Ser15 and Ser46) tumor protein p53 (p53), and meiotic recombination 11 (MRE11) protein, a member of the MRN nuclease/helicase complex, were analyzed before, during, and after etoposide-induced DNA damage. To exclude a potential influence of the chronological age of individual donors, this experiment was performed on cells from young and old donors. Our results demonstrate a decreased production of MRE11 as well as a decreased phosphorylation of ATM at Ser1981 and of p53 at Ser15 in CD8^+^CD28^−^ T cells following etoposide-induced DNA damage, while total ATM and p53 concentrations did not differ between the two CD8^+^ T-cell subsets. Interestingly, p53 phosphorylation at Ser46 was not changed in CD8^+^CD28^+^ T cells and even increased in CD8^+^CD28^−^ T cells after treatment. Identical results were obtained with cells from young and old donors demonstrating that the reduced DDR response is a general property of CD8^+^CD28^−^ T cells (Fig. 4A; no statistically significant difference between the age groups; *P* > 0.05). Data from young and elderly persons were combined for the graph shown in Fig. 4B. A schematic representation of events involved in the assembly and spreading of the DSB repair complex is depicted in Fig. 4C (Sengupta & Harris, 2005; West & van Attikum, 2006).

### DNA damage and repair in CD8^+^ T-cell subsets

**Fig. 5:**
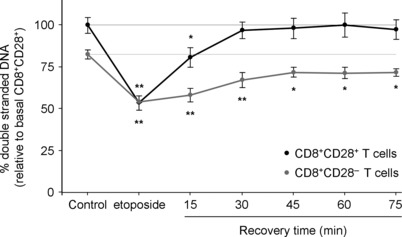
DNA damage and repair in untreated and etoposide-exposed CD8^+^ T-cell subsets. CD8^+^CD28^+^ and CD8^+^CD28^−^ T cells were either left as untreated control or exposed to 10 μg mL^−1^ etoposide for 60 min. After etoposide removal, both T-cell subsets were given time to repair DNA damage for 15, 30, 45, 60, and 75 min at 37 °C and 5% CO_2_. The figure shows mean double-stranded DNA ± SEM of six donors, independently performed and measured by an automated ‘fluorimetric detection of alkaline DNA unwinding – FADU’ assay (Moreno-Villanueva *et al.*, 2009), relative to basal CD8^+^CD28^+^ T-cell control. *P ≤ 0.05, **P ≤ 0.01 values versus respective controls (repair), and control values against each other (basal DNA damage, not indicated), *P* ≤ 0.01. While the induced DNA damage was entirely repaired after 30 min in CD8^+^CD28^+^ T cells (compared to CD8^+^CD28^+^ T-cell control, *P* = n.s.), CD8^+^CD28^−^ T cells remained unrepaired until the end of the observation period (compared to CD8^+^CD28^−^ T-cell control, *P* ≤ 0.05).

Next, DNA damage and the efficiency of DNA repair was analyzed in CD8^+^CD28^+^ and CD8^+^CD28^−^ T cells using an automated version of the FADU assay (Fig. 5). Isolated CD8^+^ T-cell subsets were either left untreated (Control) or exposed to etoposide for 60 min (Etoposide) as well as given time to repair the induced DNA damage by removing etoposide for 15, 30, 45, 60, or 75 min before analysis. Even without treatment, CD8^+^CD28^−^ T cells displayed more DNA damage than CD8^+^CD28^+^ controls. Exposure to etoposide potently induced DSBs in both cell types but the recovery time necessary to accomplish DNA repair was different in CD8^+^CD28^+^ and CD8^+^CD28^−^ T cells. While CD8^+^CD28^+^ T cells recovered in < 30 min, CD8^+^CD28^−^ T cells never reached their respective basal fluorescence level. Taken together, our results indicate that CD8^+^CD28^−^ T cells have acquired more DSBs *in vivo* and have an impaired DNA repair capacity following etoposide treatment *in vitro*.

### Etoposide-induced apoptosis in CD8^+^ T-cell subsets

**Fig. 6:**
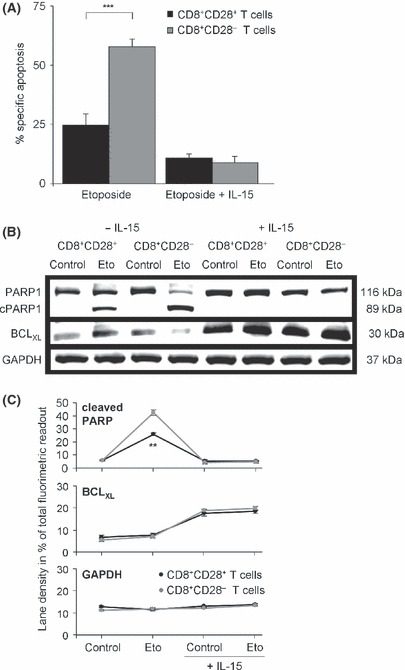
The impact of IL-15 on apoptosis and the DNA damage response in human CD8^+^CD28^+^ and CD8^+^CD28^−^ T cells. (A) Apoptotic CD8^+^CD28^+^ and CD8^+^CD28^−^ T cells after etoposide treatment. PBMCs, containing both the CD8^+^CD28^+^ and the CD8^+^CD28^−^ T-cell subset, were either treated with etoposide only (10 μg mL^−1^) for 48 h or pre-stimulated (3 h, 50 ng mL^−1^) with IL-15 before exposing them to etoposide. Bars depict specific apoptosis as measured by 7-AAD/Annexin V FACS analysis; means ± SEM; *n* = 11; ***P ≤ 0.001; CD8^+^CD28^+^ versus CD8^+^CD28^−^. (B) Apoptotic signaling under etoposide and IL-15 treatment. Isolated CD8^+^CD28^+^ and CD8^+^CD28^−^ T cells were cultured for 6 h in high-affinity 96-well plates coated with IL-15 (1 μg mL^−1^), while control experiments were performed in FCS-blocked plates in the absence of IL-15. Etoposide (10 μg mL^−1^) was then added and the cells left to incubate for another 48 h. One representative of four Western Blots, performed with cells from four individual donors, is shown. (C) The graph shows the densitometrical evaluation of grayscale values. Data represent fluorescence in individual lanes as percentages of total fluorescence in the whole blot; means ± SEM; *n* = 4; **P ≤ 0.01; CD8^+^CD28^+^ (black) versus CD8^+^CD28^−^ (gray). IL-15 treatment increased BCL_XL_ protein levels in either CD8^+^ T-cell subset compared to control; **P ≤ 0.01.

As an impaired DDR in CD8^+^CD28^−^ T cells may also lead to increased rates of apoptosis following etoposide-induced DNA damage in this cell type, we cultured PBMCs containing CD8^+^CD28^+^ and CD8^+^CD28^−^ cells with or without etoposide for 48 h. Apoptosis was assessed by Annexin V/7-aminoactinomycin D (7-AAD) staining and FACS analysis with the respective subpopulations gated. CD8^+^CD28^−^ T cells had significantly higher rates of apoptosis following exposure to etoposide than their CD8^+^CD28^+^ counterparts (Fig. 6A). To exclude a potential influence of other cell types within the PBMC fraction, this experiment was, with identical results, also performed on isolated CD8^+^CD28^+^ and CD8^+^CD28^−^ T-cell subsets (data not shown).

To test whether the homeostatic cytokine IL-15 could rescue CD8^+^CD28^−^ T-cell subsets from programmed cell death, etoposide-triggered apoptosis was analyzed in the presence of IL-15. IL-15 decreased etoposide-induced apoptosis in both CD8^+^CD28^+^ and CD8^+^CD28^−^ T cells, resulting in a similar rate of apoptosis in both T-cell subsets. IL-15 treatment caused an up-regulation of the anti-apoptotic molecule BCL_2_ like 1 (BCL_XL_), thereby preventing a cascade of signaling events that culminates in the cleavage of poly (ADP-ribose) polymerase 1 (PARP1) by active caspase 3 (Fig. 6B,C). CD8^+^CD28^−^ T cells displayed the same defects upon etoposide treatment in the altered kinetics of ATM and p53 phosphorylation in the absence and presence of IL-15 (*n* = 5; *P* > 0.05; IL-15 treated vs. untreated cells; data not shown) confirming that IL-15 did not affect DDR signaling events.

## Discussion

As CD8^+^CD28^−^ T cells have short telomeres and fail to undergo substantial proliferation following antigenic contact, this highly differentiated T-cell subset has been attributed a state of replicative exhaustion and cellular senescence (Effros *et al.*, 2005). CD8^+^CD28^−^ T cells fail to produce IL-2, are frequently oligoclonal, pro-inflammatory, and are believed to take up more immunological space, which may limit the propagation and long-term survival of other T-cell specificities (Saurwein-Teissl *et al.*, 2002). Little is still known about the molecular mechanisms that regulate survival or death of this specific cell type.

miRNA have recently been recognized to play an important role in the fine tuning of a great number of immune-regulatory responses (Baltimore *et al.*, 2008) and have also been shown to be altered in several types of ‘senescent’ cells, cells which have undergone many rounds of division, among others CD8^+^CD28^−^ T cells (Grillari & Grillari-Voglauer, 2010; Hackl *et al.*, 2010). Thus, the miRNA cluster miR-17∼92, which targets important cell cycle regulators such as p21, has been shown to be down-regulated in these cell types (Ivanovska *et al.*, 2008). We now demonstrate that the miR-23∼24∼27 cluster is up-regulated in CD8^+^CD28^−^ T cells. Recent miRNA studies on human leukemia cell lines, namely K562 cells, differentiated to megakaryocytes or erythrocytes and HL60 cells differentiated to macrophages or monocytes provided compelling evidence for an up-regulation of miR-24 that leads to a down-regulation of its target H2AX, consequently suppressing efficient DNA repair (Lal *et al.*, 2009). In view of these data on other types of highly differentiated cells, we hypothesized that the DDR of highly differentiated T cells might also be impaired.

We first analyzed whether the expression of total H2AX was altered, and did indeed find that this molecule was reduced in CD8^+^CD28^−^ T cells when compared to CD8^+^CD28^+^ T cells. To confirm that miR-24 can effectively target and suppress H2AX formation, we additionally overexpressed miR-24 in the human T-cell line Jurkat E6.1 and monitored the consecutive down-regulation of H2AX. To be able to study whether changes in miR-24 and H2AX expression were accompanied by alterations of DDR signaling events in CD8^+^CD28^−^ T cells, DNA damage was induced in isolated CD8^+^ T-cell subsets by etoposide, a classic chemotherapeutic agent that targets topoisomerase II. Indeed, CD8^+^CD28^−^ T cells failed to phosphorylate H2AX effectively and to subsequently generate nuclear γH2AX clusters. Impaired DDR signaling in CD8^+^CD28^−^ T cells was further confirmed by decreased phosphorylation of ATM (Ser1981) and of p53 (Ser15) as well as by decreased expression of MRE11 following etoposide treatment, a phenomenon that was independent of the chronological age of the donor and thus a manifestation of the intrinsic property of the respective T-cell population. The fact that p53 phosphorylation at Ser46 was not changed in CD8^+^CD28^+^ T cells and even increased in CD8^+^CD28^−^ T cells after treatment demonstrated that DDR signaling was really reduced in comparison with CD8^+^CD28^+^ T cells.

To provide definite evidence that there is a connection between miR-24 and the reduction of DDR signaling in CD8^+^CD28^−^ T cells, the overexpression or the knockdown of miR-24 in resting CD8^+^ T cells would be necessary. However, for technical reasons this experiment is not feasible because of low histone turnover in resting CD8^+^ T cells. The possibility that an attenuated DDR is independent of miR-24 can therefore not be excluded. Nevertheless, it is an intriguing hypothesis that increased miR-24 levels resulting in decreased H2AX in CD8^+^CD28^−^ T cells lead to an impairment in the spreading of DNA repair foci along the chromatin (Fig. 4C). The fact that total ATM and p53 levels were unchanged in CD8^+^CD28^−^ T cells but MRE11 expression as well as phosphorylation of ATM and p53 (Ser15) decreased in CD8^+^CD28^−^ T cells upon etoposide treatment is in favor of this possibility.

Unrepaired, clustered DNA lesions induce chromosome breakage in human cells (Asaithamby *et al.*, 2011) and can lead to permanent cell cycle arrest or apoptosis (Mandal *et al.*, 2011). Our data therefore argue in favor of an endogenous DNA repair deficiency in CD8^+^CD28^−^ T cells leading to increased apoptosis as a consequence. To test the validity of this concept, we made use of an automated FADU assay in which basal DNA damage levels as well as the DNA repair capacity following short-time exposure to etoposide were determined. *Ex vivo* isolated CD8^+^CD28^−^ T cells contained more damaged DNA than their CD8^+^CD28^+^ counterparts following isolation, but before stimulation. This pre-existing DNA damage was increased upon etoposide treatment in a similar way as in control cells of a lower differentiation stage. After 75 min recovery time, the level of initial fluorescence signal (before etoposide treatment) was not reached in CD8^+^CD28^−^ T cells suggesting a decrease in the repair capacity of these cells and consequently an accumulation of DNA damage. These results indicate that CD8^+^CD28^−^ T cells have accumulated more DNA damage upon insults *in vivo*, possibly as the result of a decreased DDR. This may increase their susceptibility to genotoxic stress, ultimately leading to increased apoptosis in this cell type. Whether ATM-independent phosphorylation of p53 (Ser46) via HIPK2 (homeodomain-interacting protein kinase-2) (D’Orazi *et al.*, 2002; Hofmann *et al.*, 2002) plays a role in the induction of apoptosis in CD8^+^CD28^−^ T cells is presently subject of further investigation. Taken together, our results suggest that CD8^+^CD28^−^ T cells are short-lived *in vivo* as they are extremely prone to die upon cellular stress.

In spite of their intrinsic susceptibility to the induction of apoptosis, CD8^+^CD28^−^ T cells still accumulate during aging or in persons with persistent viral infections (Saule *et al.*, 2006; Brunner *et al.*, 2011). This can be explained in two different ways: A continuous regeneration of this specific T-cell subset from the CD8^+^CD28^+^ T-cell pool takes place as the result of persistent antigenic stimulation. This would be the case in persons with latent CMV infection or in HIV-infected patients (Appay *et al.*, 2011). Alternatively, CD8^+^CD28^−^ T cells could survive for a prolonged period of time and be thus long-lived in specific survival supporting niches (Becker *et al.*, 2005). We have recently demonstrated that CD8^+^CD28^−^ T cells are enriched in the BM and are in close contact with IL-15-producing cells (Herndler-Brandstetter *et al.*, 2011a, b). IL-15 production is high in the human BM (Herndler-Brandstetter *et al.*, 2011a, b) and still higher in old age (Herndler-Brandstetter *et al.*, 2012). Additionally, we have shown that IL-15 strongly induces CD69 in CD8^+^CD28^−^ T cells. CD69 inhibits the expression of the sphingosine 1-phosphate receptor type 1 and lymphocyte egress from lymphoid organs (Shiow *et al.*, 2006; Maeda *et al.*, 2010). Despite their impaired DDR, CD8^+^CD28^−^ T cells display a high responsiveness to the homeostatic cytokine IL-15 (Alves *et al.*, 2003), which increases survival, rescues T-cell proliferation via the PI3K/AKT/GSK3 signaling pathway, increases anti-oxidant capacity, and improves the quality of antigen-driven responses (Alonso-Arias *et al.*, 2011; Kaur *et al.*, 2011). Our results demonstrate that IL-15 signaling enables CD8^+^CD28^−^ T cells to up-regulate the anti-apoptotic molecule BCL_XL_ and rescue them from DNA damage-induced apoptosis.

It would be interesting to investigate whether the CD8^+^CD28^−^ T cells accumulating in the BM also accumulate DNA damage and a defective DNA repair mechanism. However, owing to small sample sizes, separation of CD8^+^ T-cell subsets from BM samples is currently not possible.

In summary, our data suggest that CD8^+^CD28^−^ T cells can survive under certain circumstances in spite of their intrinsic inability to repair DNA damage and their increased capacity to undergo apoptosis. Accumulation of this specific cell type thus greatly depends on the composition of the different homing niches of an organism. This may be of particular relevance in old age or during chronic infections, both conditions for which an increased production of inflammatory as well as homeostatic cytokines is a typical feature (Franceschi *et al.*, 2000).

## Experimental procedures

### Sample preparation, purification of CD8^+^ T-cell subsets and cell culture

Peripheral blood samples were obtained from apparently healthy elderly (≥ 65 years) and young (≤ 30 years) persons who did not receive immunomodulatory drugs nor suffered from diseases known to influence the immune system, including autoimmune diseases and/or cancer. All donors had given their written informed consent to participate in this study that was approved by the Ethics Committee of Innsbruck Medical University. Peripheral blood was collected in sodium heparin tubes and PBMCs were freshly isolated by Ficoll-Hypaque (Amersham Biosciences, Amersham, UK) density gradient centrifugation. CD8^+^CD28^+^ and CD8^+^CD28^−^ T cells were enriched from PBMCs using magnetic beads (Miltenyi Biotec, Bergisch Gladbach, Germany) in a three-step MACS procedure. Briefly, CD8^+^ T cells were positively selected using anti-CD8 MultiSort microbeads and a LS column (Miltenyi Biotec). Following removal of the CD8 MultiSort microbeads, CD8^+^ T cells were incubated with anti-CD28 allophycocyanin-conjugated antibody (BD Pharmingen, Franklin Lakes, NJ, USA) and anti-allophycocyanin-conjugated microbeads. The CD8^+^CD28^−^ T-cell fraction was depleted of contaminating NK T cells using anti-CD16 microbeads. The purity of the obtained CD8^+^CD28^+^ and CD8^+^CD28^−^ T-cell populations was assessed by a FACSCanto II (BD Biosciences, Franklin Lakes, NJ, USA) and yielded population homogeneities of ≥ 90%. PBMCs and purified CD8^+^ T-cell subsets were cultured at a cell density of 2 × 10^6^ cells mL^−1^ at 37 °C in complete RPMI 1640 medium [supplemented with 10% FCS (Sigma-Aldrich, St. Louis, MO, USA) and 1% penicillin/streptomycin (Gibco)]. Experiments were performed with or without etoposide (10 μg mL^−1^) or IL-15. For the stimulation of PBMCs, soluble IL-15 was used at a concentration of 50 ng mL^−1^, whereas isolated CD8^+^ T-cell subsets were stimulated by adding them onto IL-15-coated high-affinity 96-well plates (1 μg mL^−1^; Greiner Bio-One, Frickenhausen, Germany).

### miRNA expression profiling

Two-channel microarray analysis was performed as previously described (Hackl *et al.*, 2010). In brief, locked nucleic acid probe sets (miRBase version 9.2; Exiqon, Vedbaek, Denmark), comprising 559 human miRNA as well as 77 miRPlus (intellectual property of Exiqon) sequences, were spotted on epoxy-coated Nexterion glass slides (Schott AG, Mainz, Germany). High-quality total RNA was extracted from 5 × 10^6^ CD8^+^CD28^+^ and CD8^+^CD28^−^ T cells using TRIzol reagent (Sigma-Aldrich) and Glycogen (Roche, Penzberg, Germany) as a carrier for RNA precipitation. miRNA microarray expression profiling was performed on sets of eight donor-paired CD8^+^CD28^+^ and CD8^+^CD28^−^ T-cell samples (four directly *ex vivo* and four cultured for 16 h to exclude a potential influence of cell culture on miRNA expression). First, 0.5 μg total RNA was Cy3- and Cy5-labeled with the miRCURY LNA miRNA Array labeling kit (Exiqon). RNA samples were then hybridized for 16 h to in-house spotted LNA miRNA chips using a TECAN HS 400 hybridization station (Tecan, Männedorf, Switzerland) after which the arrays were immediately scanned by a GenePix 4000B laser scanner (Axon Instruments, Foster City, CA, USA). Cy3 dye was scanned at 532 nm and Cy5 emission was recorded at 635 nm with the resolution settings adjusted to 10 μm and averaging per 1 line. Intensity values for each spot were extracted using GenePix 4.1 software and analyzed using the Bioconductor package ‘Linear models for microarray data analysis’ under R 2.9.1. In brief, the MA-transformed spot intensities of each array were background corrected (normexp algorithm) and lowess normalized (local weighted linear regression). For differential expression analysis, the signals of eight replicate spots for each miRNA per array were correlated and used for moderated hypothesis tests (moderated *t* statistic) for the contrasts of interest between pairs of CD8^+^CD28^+^ and CD8^+^CD28^−^ T cells, respectively. The resulting *P*-values were adjusted for multiple testing (*P* adj.) according to a method of Hochberg & Benjamini (1990) to control the false discovery rate (FDR). All miRNAs were then ranked in terms of their adjusted *P-*values and a cut-off of *P* ≤ 0.05 was imposed. The respective raw data as well as processed intensity data were submitted to Array Express (http://www.ebi.ac.uk/arrayexpress) according to MIAME guidelines and can be accessed under the identifiers E-MEXP-2398 and E-MEXP-3307.

### Immunofluorescence imaging

Following MACS separation, isolated CD8^+^ T-cell subsets were washed and equilibrated in complete RPMI 1640 medium at a cell density of 2 × 10^6^ mL^−1^ for 1 h at 37 °C. Two hundred microlitres of cell suspension aliquots, containing 4 × 10^5^ cells, were treated with 10 μg mL^−1^ etoposide to induce DSBs for 30 min at 37 °C while untreated cells were used as control. Following the indicated incubation period, CD8^+^ T-cell subsets were immediately fixed by adding 200 μL CytoFix/Cytoperm solution (BD Pharmingen) for 15 min at 37 °C. After a subsequent washing step in 1× PermWash buffer (BD Pharmingen), CD8^+^ T-cell subsets were permeabilized for 30 min on ice in PhosFlow Perm Buffer III (BD Pharmingen). Following another washing step in 1× PermWash buffer, CD8^+^ T-cell subsets were incubated in 100 μL of a primary antibody solution containing 1:400 mouse anti-γH2AX phospho-Ser139 (Abcam, Cambridge, UK) in PermWash buffer for 1 h at 37 °C. Thereafter, washing in 1× PermWash buffer was followed by a 45-min incubation period at RT in 100 μL of a secondary solution containing 1:200 rabbit anti-mouse AlexaFlour488-conjugated Ab (Abcam) and 1:50 TO-PRO solution (Invitrogen, Carlsbad, CA, USA) in 1× PermWash buffer. After washing in 1× PermWash buffer twice, cells were resuspended in small droplets of glycerol, the cell suspension carefully mixed, transferred onto microscope slides, and covered with coverslips. Cells were given time to settle into the same layer by storing the slides in a vertical position for 3 h at 4 °C. Immunofluorescence images were taken using a μ-Radiance scan head (Bio-Rad Laboratories, Hercules, CA, USA) attached to a Zeiss Axiophot confocal scanning microscope system and Zeiss Plan-Neofluar objective lenses at 40×/0.75 operated at RT and subsequently analyzed by Zeiss LaserSharp 2000 software (Carl Zeiss, Oberkochen, Germany). Experiments were performed in duplicates for each CD8^+^ T-cell subset and independently validated for four donors. To quantify γH2AX spot formation, the samples were screened microscopically by two independent persons and the number of cells with clusters in their nuclei as well of the number of spots per nucleus was determined. 200 cells per sample were analyzed.

### Transfection of Jurkat E6.1 cells with miR-24

As for technical reasons neither overexpression nor knockdown of miRNAs is possible in resting CD8^+^CD28^+^ and CD8^+^CD28^−^ T cells, we overexpressed miR-24 in the human leukemic T-cell lymphoblast line Jurkat E6.1. Jurkat cells were cultured at a cell density of 0.5 to 3 × 10^6^ cells mL^−1^ in a humidified incubator operated at 37 °C and 5% CO_2_ in complete RPMI 1640 medium (supplemented with 10% FCS and 1% penicillin/streptomycin) and passaged as required to maintain logarithmic growth. One hour prior to transfection, 2 × 10^5^ Jurkat cells in 100 μL medium containing 20% FCS were transferred into 24-well plates and given time to equilibrate for the indicated time period. 750 ng (3 μL of 20 μm stock) miR-24 miScript miRNA mimic (Qiagen, Hilden, Germany) was diluted in 100 μL medium without serum or antibiotics before adding 4 μL Attractene transfection reagent (Qiagen). Following an incubation period of 15 min to allow the formation of transfection complexes at room temperature, this suspension was added to the cells in a ratio of 1:1 and cultured for 6 h at 37 °C. Another 400 μL medium containing 10% FCS and antibiotics were added then, finally yielding a miRNA concentration of 100 nm. Twenty-four hour post-transfection 400 μL fresh medium was added and the cells further cultured for another 3 days before analysis. Control Jurkat cells were treated equally but were ‘transfected’ with medium alone, were mock-transfected using empty complexes (Mock), or were transfected with nonsense miRNA (Scrambled; Qiagen).

### Western Blotting

Whole cell protein lysates (TNE buffer: 50 mm Tris HCl pH 8.0, 150 mm NaCl, 0.5 mm EDTA, and 1% TritonX100, supplemented with protease and phosphatase inhibitors) of 2 × 10^6^ CD8^+^ T cells/Jurkat cells per described time point and treatment were subject to Western Blot analysis (denaturing gel electrophoresis on 4–20% gradient Tris-Glycine precast polyacrylamide gels; Thermo Scientific, Waltham, MA, USA) and tested for the expression of total H2AX, phospho-Ser1981 and total ATM, phospho-Ser15, phospho-Ser46 and total p53, MRE11, BCL_XL_, PARP1/cleaved PARP1, and GAPDH using primary antibodies (Abcam and Cell Signaling, Danvers, MA, USA). Protein bands were visualized using horseradish-peroxidase-conjugated secondary Abs, ECL Western Blot substrate, or SuperSignal Western Femto substrate (all Thermo Scientific), depending on the obtained signal strength, and an Alpha Innotech chemiluminescence detection unit with AlphaEaseFC software (Biozym, Hessisch Oldendorf, Germany). For H2AX expression in CD8^+^ T-cell subsets, densitometrical evaluation of obtained grayscale values normalized to GAPDH was performed and plotted as fold-regulation relative to CD8^+^CD28^−^ T cells.

### Automated fluorimetric detection of alkaline DNA unwinding

We assessed the efficiency of DNA repair in CD8^+^CD28^+^ and CD8^+^CD28^−^ T cells by a recently developed, new generation automated version of the fluorimetric detection of alkaline DNA unwinding (FADU) assay (Moreno-Villanueva *et al.*, 2009). This technique is based on progressive DNA unwinding (denaturation) under highly controlled conditions of alkaline pH, time, and temperature. The starting points for the unwinding process are not only DSBs induced by reactive oxygen species (ROS), irradiation, or chemical compounds, but also replication forks or chromosome ends. The dye SYBR Green (Invitrogen) intercalates into double-stranded DNA, essentially leaving stretches of unwound DNA unaffected. The loss of the resulting fluorescence signal, compared to untreated controls, is proportional to the amount of DSBs. The FADU assay is performed under extreme alkaline conditions that interfere with protein-protein interactions and thus dissolve T and D loops present at DNA caps, which allows comparing samples with different telomere lengths. Isolated CD8^+^ T-cell subsets were either left untreated or exposed to 10 μg mL^−1^ etoposide for 60 min as well as given time to repair the induced DNA damage after removal of etoposide for 15, 30, 45, 60, or 75 min, respectively, before performing the automated FADU assay as described elsewhere (Moreno-Villanueva *et al.*, 2009). Samples were analyzed in a 96-well plate fluorescence reader at 492 nm excitation/520 nm emission immediately after SYBR Green addition. Experiments were performed with three technical replicates per sample and the average calculated. Values from six paired samples of CD8^+^CD28^+^ and CD8^+^CD28^−^ T cells obtained from six individual donors, performed on three independent runs, were plotted onto a scale representing means of double-stranded DNA compared to basal CD8^+^CD28^+^ T-cell levels. The recovery times after DNA damage were calculated using two-tailed Student’s *t*-test.

### Quantitative RT–PCR

Total RNA was extracted from untreated and etoposide-treated CD8^+^CD28^+^ and CD8^+^CD28^−^ T cells/Jurkat cells and cDNA was synthesized by applying the miRCURY LNA Universal real-time (RT) system (Exiqon) for evaluation of miR-24 expression. Quantitative RT–PCR experiments were performed using the LightCycler II 480 System (Roche Diagnostics, Risch, Switzerland) and GAPDH (fwd: GAGTCAACGGATTTGGTCGT, rvs: GATCTCGCTCCTGGAAGATG) or U6 miRNA as housekeeping genes for normalization purposes and the relative quantification of target genes. The amplification protocol used was as follows: initial incubation at 95 °C for 8 min followed by 40 amplification cycles at 95 °C for 10 s and 60 °C for 60 s according to the manufacturer’s instructions. Quality control of amplified gene targets was performed by assessing the melting peak profiles of obtained PCR products. GAPDH normalized threshold cycle (CT) values (ΔCT = CT_gene_ − CT_GAPDH_) were used to calculate the relative gene expression compared to control samples (2^−ΔΔCT^).

### Assessment of apoptosis by Annexin V/7-AAD staining

Viable cells were determined counting Annexin V/7-AAD double-negative cells by FACS analysis (BD FACS Canto II with BD FACS Diva software, 40 000 events analyzed). Experiments were performed on PBMCs, counterstained with anti-CD28-APC, anti-CD3-APC-Cy7, and anti-CD8-PE (BD Pharmingen) as well as on isolated CD8^+^ T-cell subsets. Percent specific apoptosis over background was calculated by normalizing against respective controls.
